# NDUFA4L2 is associated with clear cell renal cell carcinoma malignancy and is regulated by ELK1

**DOI:** 10.7717/peerj.4065

**Published:** 2017-11-17

**Authors:** Lei Wang, Zhiqiang Peng, Kaizhen Wang, Yijun Qi, Ying Yang, Yue Zhang, Xinyuan An, Shudong Luo, Junfang Zheng

**Affiliations:** 1 Department of Urology, Beijing Friendship Hospital, Capital Medical University, Beijing, China; 2 Department of Biochemistry and Molecular Biology, Capital Medical University, Beijing, China; 3 Core Facilities Center, Capital Medical University, Beijing, China; 4 Key Laboratory of Biology of Insect-Pollinator, Ministry of Agriculture, Institute of Apicultural Research, Chinese Academy of Agricultural Sciences, Beijing, China; 5 Beijing Key Laboratory for Tumor Invasion and Metastasis, Cancer Institute of Capital Medical University, Beijing, China

**Keywords:** NDUFA4L2, Clear cell renal cell carcinoma, Transcription factor, ELK1

## Abstract

**Background:**

Clear cell renal cell carcinoma (ccRCC) is the most common and lethal cancer of the adult kidney. However, its pathogenesis has not been fully understood till now, which hinders the therapeutic development of ccRCC. NADH dehydrogenase (ubiquinone) 1 alpha subcomplex 4-like 2 (NDUFA4L2) was found to be upregulated and play an important role in ccRCC. We aimed to further investigate the underlying mechanisms by which NDUFA4L2 exerted function and its expression level was upregulated.

**Methods:**

The Gene Expression Omnibus (GEO) and The Cancer Genome Atlas (TCGA) data were mined to verify the change of NDUFA4L2 expression level in ccRCC tissues. The correlation between expression level of NDUFA4L2 and cell proliferation/apoptosis was explored by Gene Set Enrichment Analysis (GSEA). Protein-protein interaction (PPI) network of NDUFA4L2 was constructed. Biological process and involved pathways of NDUFA4L2 were analyzed by gene ontology (GO) and the Kyoto Encyclopedia of Genes and Genomes (KEGG) pathway. The transcription factors (TFs) which can induce the expression of NDUFA4L2 were explored in clinical samples by correlation analysis and its regulation on the expression of NDUFA4L2 was verified by knockdown experiment.

**Results:**

NDUFA4L2 was verified to be overexpressed in ccRCC tissues and its expression level was increased accordingly as the American Joint Committee on Cancer (AJCC) stage progressed. A high NDUFA4L2 level predicted the poor prognosis of ccRCC patients and correlated with enhanced cell proliferation and anti-apoptosis. NDUFA4L2 may interact with 14 tumor-related proteins, participate in growth and death processes and be involved in ccRCC-related pathways, such as insulin-like growth factor 1 (IGF-1), mammalian target of Rapamycin (mTOR) and phosphoinositide 3 kinase serine/threonine protein kinase (PI3K/AKT). ETS domain-containing protein ELK1 level positively correlated with the level of NDUFA4L2 in ccRCC tissues and ELK1 could regulate the expression of NDUFA4L2 in ccRCC cells.

**Discussion:**

NDUFA4L2 upregulation was associated with ccRCC malignancy. NDUFA4L2 expression was regulated by ELK1 in ccRCC cells. Our study provided potential mechanisms by which NDUFA4L2 affected ccRCC occurrence and progression.

## Introduction

Renal cell carcinoma (RCC) is the third most common urological cancer with the highest mortality rate (Bukowski, 2009; Yang et al., 2010). Clear cell RCC (ccRCC) accounts for approximately 80% of all primary malignant kidney tumors (Karumanchi, Merchan & Sukhatme, 2002; Rini, Campbell & Escudier, 2009). However, the underlying mechanisms of ccRCC occurence and progression remain unclear, which retarded the development of effective diagnostic and therapeutic targets.

It was reported that NADH dehydrogenase (ubiquinone) 1 alpha subcomplex 4-like 2 (NDUFA4L2) was highly expressed at both mRNA and protein levels in ccRCC samples but undetectable in normal kidney tissue samples. NDUFA4L2 mRNA expression level correlated with tumor stage and overall survival (Minton et al., 2016), and overexpression of NDUFA4L2 was also reported to associate with poor prognosis in ccRCC patients (Lv et al., 2016). Furthermore, knockdown of NDUFA4L2 impaired cell proliferation in hypoxia by increasing mitochondrial reactive oxygen species (ROS) generation in cultured RCC cells (Liu et al., 2016; Lv et al., 2016; Minton et al., 2016). However, the underlying mechanism by which NDUFA4L2 exerted function and NDUFA4L2 expression level was upregulated need to be further investigated.

Tumor proliferation plays an important role in tumor development and is controlled by dynamic adjustments in transcriptional networks (Xue et al., 2012). The aberrant expression of tumor-related genes is triggered at multiple levels including copy number variation, gene mutation, abnormal levels of methylation, miRNAs, transcription factors and post-translational modification, etc (Fan et al., 2017; Kowalczyk et al., 2016; Pronina et al., 2017; Xiao et al., 2013). Of them, transcription factors play the prominent regulatory role (Doghman et al., 2013). However, in clinical samples, regulatory mechanisms underlying NDUFA4L2 overexpression was not fully understood so far.

In the present study, we verified the previous findings that NDUFA4L2 was significantly upregulated in ccRCC tissues compared to adjacent normal tissues. A high level of NDUFA4L2 correlated with the progression and poor prognosis of ccRCC and was associated with proliferation and apoptosis in ccRCC patients. The NDUFA4L2 level also positively correlated with ETS domain-containing protein ELK1 expression in ccRCC tissues and was regulated by ELK1 in ccRCC cells.

## Materials and Methods

### Gene expression and clinical data in GEO and TCGA database

The microarray data from Gene Expression Omnibus (accession number GSE6344) and mRNA data (RNA Seq v2) from The Cancer Genome Atlas (TCGA) were used. TCGA_KIRC IlluminaHiSeq_RNASeqV2 (Synapse ID: syn2320105) data were obtained from https://www.synapse.org/#!Synapse:syn2320105. The NDUFA4L2, nuclear factor kappa-B1 (NF-κB1), REL proto-oncogene nuclear factor-κB p65 subunit (REL), ELK1, ever shorter telomeres 1 (EST1) and hypoxia inducible factor-1α (HIF1α) mRNA levels were used in this study. Clinical Kidney Renal Clear Cell Carcinoma (TCGA, Nature 2013) data were downloaded from cBioPortal database (http://www.cbioportal.org/study?id=kirc_tcga_pub).

### Gene set enrichment analysis

The association between expression level of NDUFA4L2 and biological processes was analyzed using Gene Set Enrichment Analysis (GSEA v2.2, http://www.broad.mit.edu/gsea/). GSEA calculates a gene set Enrichment Score (ES) that estimates whether genes from pre-defined gene set (obtained from the Molecular Signatures Database, MSigDB) are enriched in the NDUFA4L2 high/low expression group or distributed randomly. Thresholds for significance were determined by permutation analysis (1,000 permutations). False Discovery Rate (FDR) was calculated. A gene set is considered significantly enriched when the FDR score is < 0.25 (Subramanian et al., 2005).

### Protein-protein interaction (PPI) network construction

Search Tool for the Retrieval of Interacting Genes/Proteins (IntAct; https://www.ebi.ac.uk/intact/?conversationContext=2) is a database of known and predicted protein interactions that may aid the comprehensive description of cellular mechanisms and functions. The PPI network of NDUFA4L2 was constructed using the IntAct database. Experimental result evidences about the interaction among proteins were downloaded from the BioGRID database (https://thebiogrid.org/).

### GO and KEGG pathway analyses

To explore the functional annotation enrichment of NDUFA4L2 and its binding partners in ccRCC, Gene Ontology (GO) and unifying functional Kyoto Encyclopedia of Genes and Genomes (KEGG) pathways were analyzed in WebGestalt (http://www.webgestalt.org/).

### Prediction of TFs associated with binding motifs of NDUFA4L2

Transcription Factors (TFs) bound specifically to their corresponding binding motif (BMo) (Srivastava et al., 2014) and regulated the expression of their target genes. Potential TFs of NDUFA4L2 gene in ccRCC were predicted by Gene Regulation program (P Match 1.0 and Match 1.0; http://gene-regulation.com/pub/programs.html). The JASPAR public database was used to verify the binding sites of these TFs (Mathelier et al., 2016).

### Cell culture, ELK1 knockdown and Western blotting

Human ccRCC 786-O (Williams et al., 1978) and ACHN (Kochevar, 1990) cell lines (American Type Culture Collection, ATCC, Manassas, VA, USA) were grown in RPMI 1640 medium and Dulbecco’s modified Eagle’s medium (DMEM) (both from Gibco, Waltham, MA, USA), respectively. These media contain 10% fetal bovine serum (FBS, Hyclone, Logan, UT, USA) and 1% antibiotic-antimycotic agent (Life Technologies, Inc., Grand Island, NY, USA). Cells were grown at 37 °C and 5% CO_2_.

Small interfering RNA (siRNA) duplexes pools directed against ELK1 (sc-35290) and control scrambled RNAi (sc-37007) were bought from Santa Cruz Biotechnology (Santa Cruz, CA, USA). Cells were grown to 80% confluency in 35-mm dishes, transfected with 2 µl Lipofectamine 2000 (Invitrogen, Carlsbad, CA, USA), and mixed with 30 pmol of the synthetic ELK1 siRNA pool. The cells were then harvested and analyzed after 48 h of transfection.

The primary antibodies specific for ELK1 was bought from Santa Cruz, NDUFA4L2 and GAPDH antibodies were purchased from Abcam (Cambridge, MA, USA).

### Statistical analysis

Paired and unpaired samples were analyzed by paired and independent sample *t*-test, respectively. The relationship between the NDUFA4L2 expression level and clinical stages was analyzed by one-way analysis of variance (ANOVA). Receiver operator characteristic (ROC) curve analysis was applied to detect the optimal cutoff point discriminating ccRCC and normal tissues. The Log rank test for the generated Kaplan–Meier (KM) curve was conducted to evaluate the association between the expression level of NDUFA4L2 and survival rate. The correlations of NDUFA4L2 and transcription factor expression levels were analyzed by Pearson correlation. Statistical analyses were performed using the SPSS 19.0 (SPSS Inc, Chicago, IL, USA) and Graphpad Prism 5 (Graphpad software Inc, San Diego, CA, USA). *p* < 0.05 was considered to be statistically significant.

## Results

### NDUFA4L2 is overexpressed and correlates with poor prognosis in ccRCC

**Figure 1:**
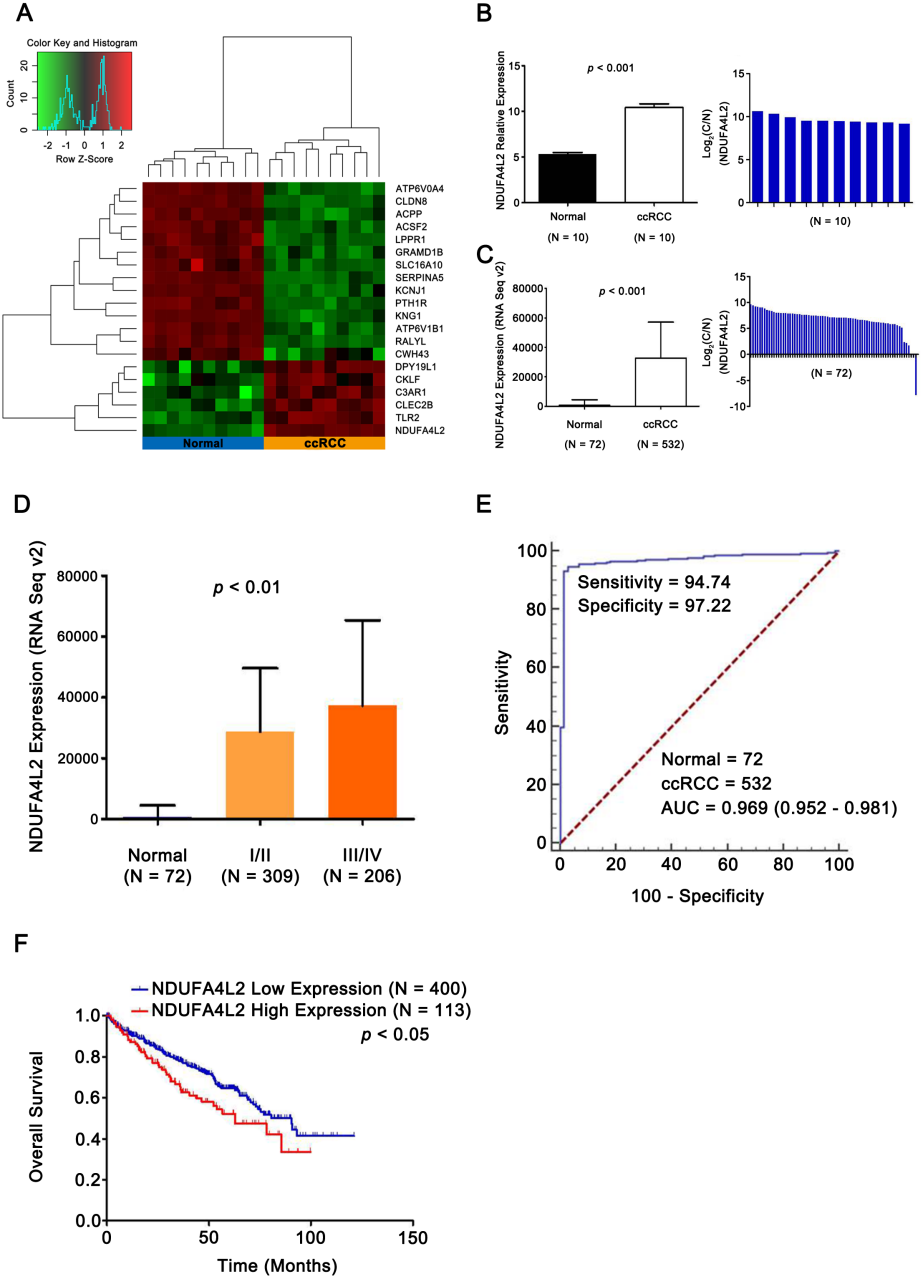
NDUFA4L2 is overexpressed and correlates with poor prognosis in ccRCC. (A) Heatmap showing differential expression of 20 top genes. (B–C) Compared with adjacent nontumor tissues, expression level of NDUFA4L2 in ccRCC tissues was upregulated. Log2(C/N) represents Log2(NDUFA4L2 level in ccRCC (C) tissues/NDUFA4L2 level in adjacent normal (N) tissues). (D) Expression level of NDUFA4L2 in each ccRCC AJCC stage of TCGA_KIRC was upregulated. (E) ROC curve results showed that the NDUFA4L2 level could discriminate normal and ccRCC tissues. (F) KM overall survival curve according to the median value of the NDUFA4L2 expression level in ccRCC patients.

To identify differentially expressed genes related with ccRCC occurrence and development, 10 pairs of ccRCC and matched normal samples in GEO database (GSE6344) were analyzed. 1,549 differentially expressed genes were identified (*p* < 0.05). Expression heatmap of top 20 genes was constructed by R 3.2.0 (R Core Team, 2015) and NDUFA4L2 was the most prominently upregulated gene (Figs. 1A and 1B). Analysis results in TCGA dataset further validated that the expression level of NDUFA4L2 was abnormally upregulated in ccRCC tissues compared with normal tissues and matched adjacent nontumor tissues (Fig. 1C). The expression level of NDUFA4L2 was increased accordingly as the American Joint Committee on Cancer (AJCC) stage progressed (Fig. 1D). The NDUFA4L2 expression level could discriminate between ccRCC and normal tissues with an area under the ROC curve of 0.969 (Fig. 1E) and was correlated with prognosis in ccRCC (Fig. 1F). The KM curve revealed that patients with a high NDUFA4L2 expression level presented lower overall survival probability than those with a low NDUFA4L2 expression level. These results indicated that upregulated NDUFA4L2 level in ccRCC tissues was correlated with the tumorigenesis, development and poor prognosis of ccRCC.

### NDUFA4L2 is positively correlated with cell proliferation and anti-apoptosis in ccRCC

**Figure 2:**
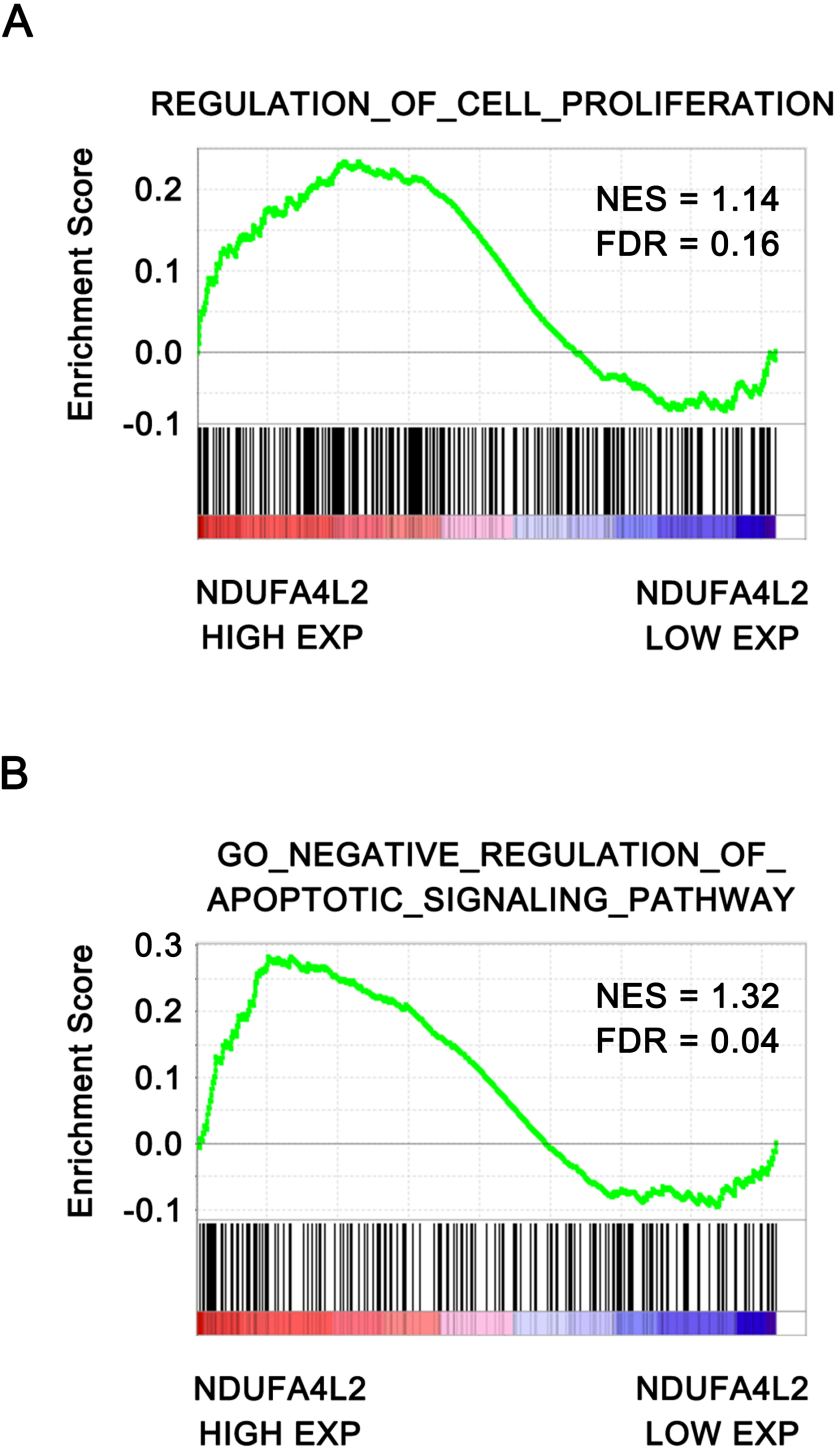
NDUFA4L2 level is positively correlated with cell proliferation and anti-apoptosis. The ccRCC patients in the TCGA_KIRC dataset were divided into high and low NDUFA4L2 expression groups according to the median value of NDUFA4L2 expression level. Enrichment of gene sets was performed by GSEA.

To further verify that NDUFA4L2 was related with ccRCC tumorigenesis, the correlation of NDUFA4L2 expression level with cell proliferation and apoptosis were analyzed by GSEA. The ccRCC patients in TCGA dataset were divided into high and low NDUFA4L2 expression groups according to the median value of the NDUFA4L2 level. Gene sets of cell proliferation and negative regulation of apoptotic signaling pathway were highly enriched in the high NDUFA4L2 expression group (Figs. 2A and 2B, FDR < 0.25). These data suggested that a high NDUFA4L2 expression level correlated with enhanced cell proliferation and decreased apoptosis.

### NDUFA4L2 is involved in multiple types of cancer-related biological processes and pathways

**Figure 3:**
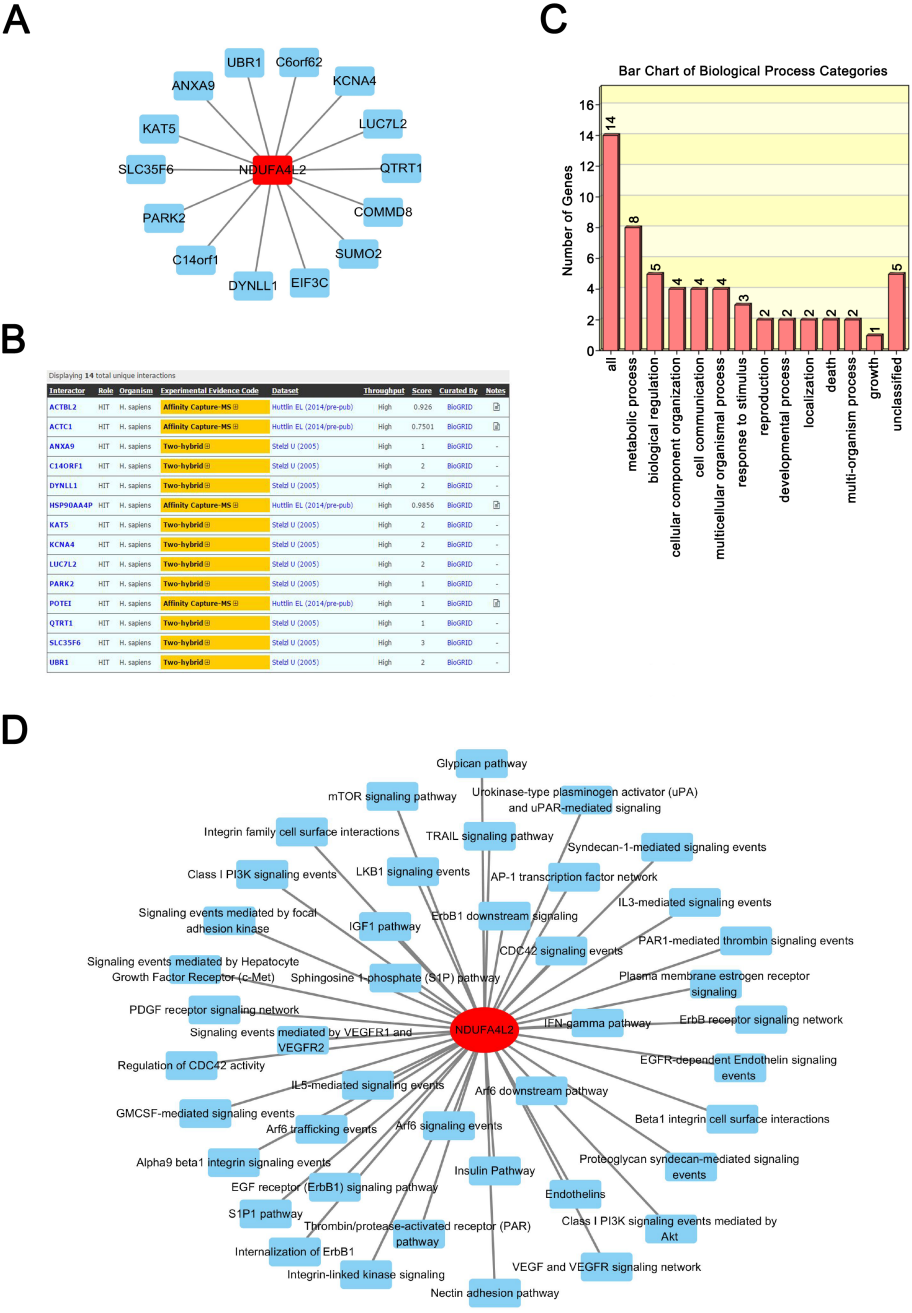
NDUFA4L2 is involved in multiple types of cancer-related biological processes and pathways. (A–B) Protein-Protein Interaction (PPI) network analysis and experimental evidence for NDUFA4L2. (C) GO biological process analysis of NDUFA4L2 and its 14 binding proteins. (D) KEGG analysis for NDUFA4L2 and its binding partners.

Proteins exert their functions via interacting proteins and involved pathways. To gain insight into the underlying mechanism by which NDUFA4L2 is involved in ccRCC development, we performed Protein-Protein Interaction (PPI), GO and KEGG pathway analyses to find out the interacting proteins and involved pathways of NDUFA4L2. PPI analysis showed 14 proteins could interact with NDUFA4L2 (Fig. 3A) and these interactions were experimentally verified by affinity capture-mass spectrometry (MS) and two-hybrid assays (https://thebiogrid.org/121231/summary/homo-sapiens/ndufa4l2.html, Fig. 3B). These proteins participated in 13 significant biological processes, of which one was related to death and two were involved in growth, indicating a potential correlation of NDUFA4L2 with ccRCC tumorigenesis and development (Fig. 3C). NDUFA4L2 and its binding partners were involved in multiple types of ccRCC-related signaling pathways, such as the insulin-like growth factor 1 (IGF-1) signaling pathway, the mammalian target of Rapamycin (mTOR) signaling pathway, and the phosphoinositide 3 kinase serine/threonine protein kinase (PI3K/AKT) signaling pathway (Fig. 3D). It was known that IGF-1 pathway played an important role in cell proliferation and apoptosis resistance in RCC (Tracz et al., 2016). Hence, NDUFA4L2 might exert its functions via these binding partners and associated pathways.

**Figure 4:**
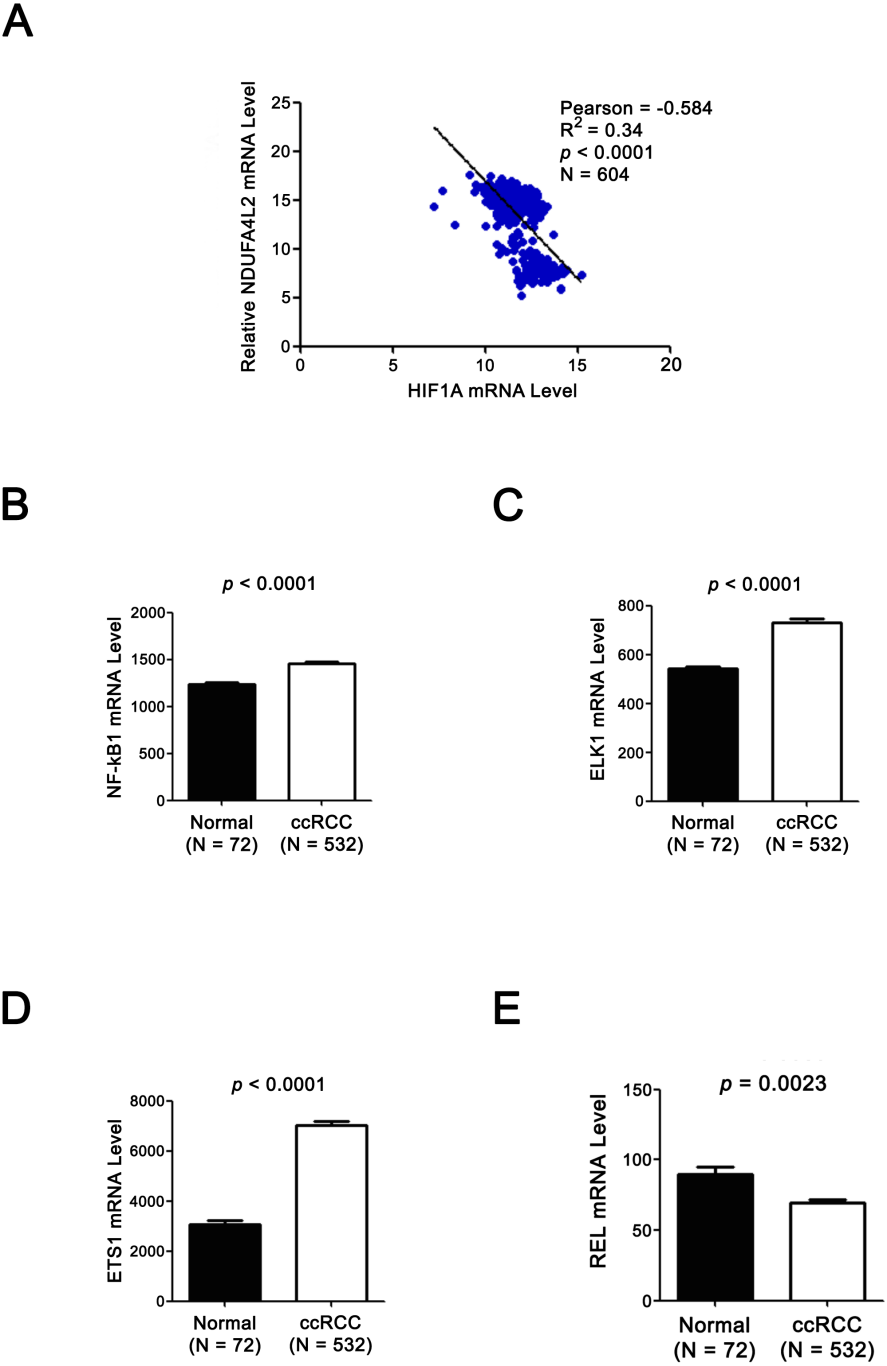
Expression levels of TF HIF1*α* and NDUFA4L2 are not positively correlated and three other TFs of NDUFA4L2 are upregulated in ccRCC. (A) Expression level of TF HIF1α was not positively correlated with the level of NDUFA4L2 in TCGA dataset. (B–E) TF NF-kB1, ELK1 and ETS1, but not REL levels were upregulated in ccRCC. The expression levels of TFs in normal and ccRCC samples were downloaded from TCGA_KIRC dataset. Independent sample *t*-test was used.

### The expression levels of NDUFA4L2 and ELK1 are positively correlated in ccRCC tissues and ELK1 regulates the expression of NDUFA4L2 in ccRCC cells

**Figure 5:**
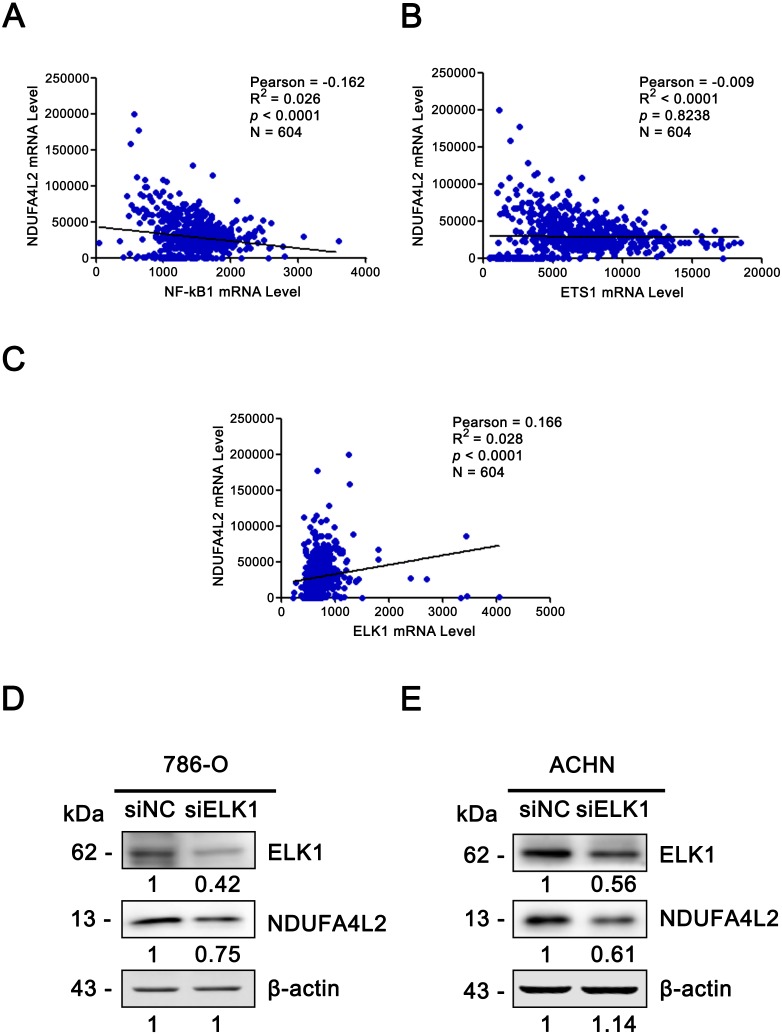
ELK1 regulated the expression of NDUFA4L2. (A–C) TF ELK1, but not NF-kB1 and ETS1 level was positively correlated with NDUFA4L2 level in ccRCC. The mRNA levels of TFs were downloaded from TCGA_KIRC dataset. Pearson correlation analysis was used. (D–E) Western blotting result showed the protein level of NDUFA4L2 was downregulated with the knockdown of ELK1.

Since the upregulation of NDUFA4L2 expression level played the important role in ccRCC occurrence and development, it is necessary to explore the reasons why the expression level of NDUFA4L2 was increased in ccRCC. In genomic level, gene copy number amplification and gene mutation are one of the reasons for the upregulation of gene expression level. However, bioinformatics analysis results revealed that copy number variation and mutation for NDUFA4L2 gene were not the reasons for NDUFA4L2 level upregulation in ccRCC (Fig. S1). Transcriptional regulation often affects gene expression at transcriptional level and the transcription factors (TFs) play important roles in this process. Hence, we paid attention to the TFs which could regulate the expression of NDUFA4L2. Unexpectedly, the level of reported TF HIF1α was not positively correlated with the level of NDUFA4L2 in the TCGA dataset (Fig. 4A), implying that there were unidentified TFs correlating with the level of NDUFA4L2. NF-κB1, ELK1, ETS1 and REL were predicted TFs of NDUFA4L2 by gene regulation program and were expressed in ccRCC patients. Of them, NF-κB1, ELK1 and ETS1, but not REL expression levels were significantly upregulated in ccRCC (all *p* < 0.01) compared with normal renal tissues (Figs. 4B–4E). JASPAR database analysis results also revealed these three TFs could bind to the promoter region of NDUFA4L2. To further verify the correlation of the NDUFA4L2 expression level with the levels of these three TFs from a clinical aspect, the Pearson correlation analysis between expression levels of NF-κB/ELK1/ETS1 and NDUFA4L2 was done in ccRCC tissues. Results showed that the ELK1 was the only transcription factor positively correlated with the expression level of NDUFA4L2 (Figs. 5A–5C).

Since the correlation between the expression of NDUFA4L2 and ELK1 is very weak, we further performed the knockdown of ELK1 and detected the levels of NDUFA4L2 in ccRCC cells 786-O and ACHN to elucidate the direct regulatory role of ELK1 on NDUFA4L2 expression. Results showed that with the knockdown of ELK1, the protein level of NDUFA4L2 was downregulated, verifying that the levels of NDUFA4L2 could be regulated by ELK1 expression (Figs. 5D–5E).

## Discussion

In the present study, both GEO and TCGA database analysis results confirmed that the expression level of NDUFA4L2 was significantly upregulated in ccRCC tissues. The NDUFA4L2 level increased accordingly as the AJCC stage progressed. A high level of NDUFA4L2 predicted poor clinical outcome of ccRCC patients and correlated with enhanced cell proliferation and anti-apoptosis. NDUFA4L2 was involved in multiple cancer-related growth and survival pathways and its expression level was positively correlated with ELK1 in ccRCC tissues. This study supports the findings of a recent report (Minton et al., 2016). In addition, we add the new findings which reveal that NDUFA4L2 could interact with 14 binding partners to participate in multiple types of biological pathways, ELK1 could regulate the expression of NDUFA4L2. Interestingly, it was observed that the expression of HIF1α did not correlate with the expression of NDUFA4L2 in clinical samples.

NDUFA4L2 was aberrantly expressed in multiple types of cancer, including malignant hepatocellular carcinoma (Lai et al., 2016), colorectal cancer (Lv et al., 2016), ccRCC (Lv et al., 2016). Inactivation of NDUFA4L2 increased mitochondrial activity and oxygen consumption, resulting in ROS accumulation and apoptosis in hepatocellular carcinoma (Lai et al., 2016). Induction of the mitochondrial NDUFA4L2 protein decreased oxygen consumption by inhibiting complex I activity in fibroblast and HeLa cell lines and mice (Tello et al., 2011). Overexpression of NDUFA4L2 was also reported to be associated with poor prognosis in colorectal cancer patients (Lv et al., 2016). In ccRCC cell lines, NDUFA4L2 knockdown impaired cell proliferation and colony formation (Minton et al., 2016). NDUFA4L2 was also associated with poor prognosis in ccRCC (Lv et al., 2016). This study comprehensively explored the function of NDUFA4L2 in ccRCC and further found NDUFA4L2 promoted cell proliferation and inhibited apoptosis in clinical level using GSEA.

NDUFA4L2 was reported to promote cell proliferation in hypoxia by increasing mitochondrial ROS and nucleic acid generation (Minton et al., 2016). In this study, we found most of its binding partners were associated with tumor occurrence and development (Brown et al., 2016; Starokadomskyy et al., 2013). NDUFA4L2 and its binding partners participated in growth and death processes and multiple cancer-related KEGG pathways, such as the IGF-1 (Tracz et al., 2016), mTOR (Populo, Lopes & Soares, 2012) and PI3K/AKT signaling pathways (Guo et al., 2015). IGF-1 enhanced transforming growth factor-β (TGF-β) signaling and raised IGF-binding protein 3 (IGFBP-3) levels which had growth-promoting effect in RCC (Rosendahl & Forsberg, 2006). mTOR and AKT signaling play well-known roles in ccRCC. Hence, NDUFA4L2 may promote cell proliferation and anti-apoptosis through multiple ccRCC-related signaling pathways.

It was reported that NDUFA4L2 was induced by TF HIF1α (Minton et al., 2016; Tello et al., 2011). However, their work was done in cellular and animal (mice) level. In this study, we analyzed the correlation between expression levels of HIF1α and NDUFA4L2 in clinical samples. Unexpectedly, we found that the HIF1α expression level was negatively correlated with the NDUFA4L2 expression level, which was inconsistent with the conclusion that NDUFA4L2 was induced by TF HIF1α and imply that other factors may regulate NDUFA4L2 expression. Pearson correlation analysis of clinical samples showed the expression level of TF ELK1 was positively correlated with the expression level of NDUFA4L2 and ELK1 acted as a potential TF which could induce NDUFA4L2 expression in ccRCC samples. The ELK1 knockdown experiment validated the regulation of ELK1 on NDUFA4L2 expression. The abnormal activation of ELK1 played an important role in the malignant transformation of prostate cancer (Patki et al., 2013). This study further verified that the important role of ELK1 in ccRCC occurrence and development.

In conclusion, NDUFA4L2 upregulation was associated with ccRCC malignancy and was regulated by ELK1 in ccRCC. Our study provided potential mechanisms by which NDUFA4L2 affected ccRCC occurrence and progression.

##  Supplemental Information

10.7717/peerj.4065/supp-1 Figure S1 NDUFA4L2 gene copy number amplification and gene mutation are not the reasons of NDUFA4L2 level upregulation (A–B) Analysis result from cBioPortal TCGA_KIRC dataset showed that there were no mutations or copy number amplification for NDUFA4L2 gene in ccRCC. (C) The OncoPrint from cBioPortal showed that NDUFA4L2 genetic alterations occurred in 3% (17/499) ccRCC cases. However, these genetic alteration of NDUFA4L2 did not lead to the upregulation of NDUFA4L2 mRNA level. (D) Compared with normal copy number samples (diploid), copy number gain samples did not lead to the significant increase of NDUFA4L2 mRNA level.

## Additional Information and Declarations

### Data Availability

The following information was supplied regarding data availability: The research in this article did not generate any data or code. Our raw data [TCGA_KIRC, IlluminaHiSeq_RNASeqV2, Synapse ID: syn2320105 and Kidney Renal Clear Cell Carcinoma (TCGA, Nature 2013)] were downloaded from https://www.synapse.org/#!Synapse:syn2320105 and http://www.cbioportal.org/study?id=kirc_tcga_pub, respectively.
