# Supplementary figures and images for: NDUFA4L2 is associated with clear cell renal cell carcinoma malignancy and is regulated by ELK1
Source: PeerJ. 2017 Nov 17;5:e4065. doi: 10.7717/peerj.4065 (PMC5695248; doi:10.7717/peerj.4065)

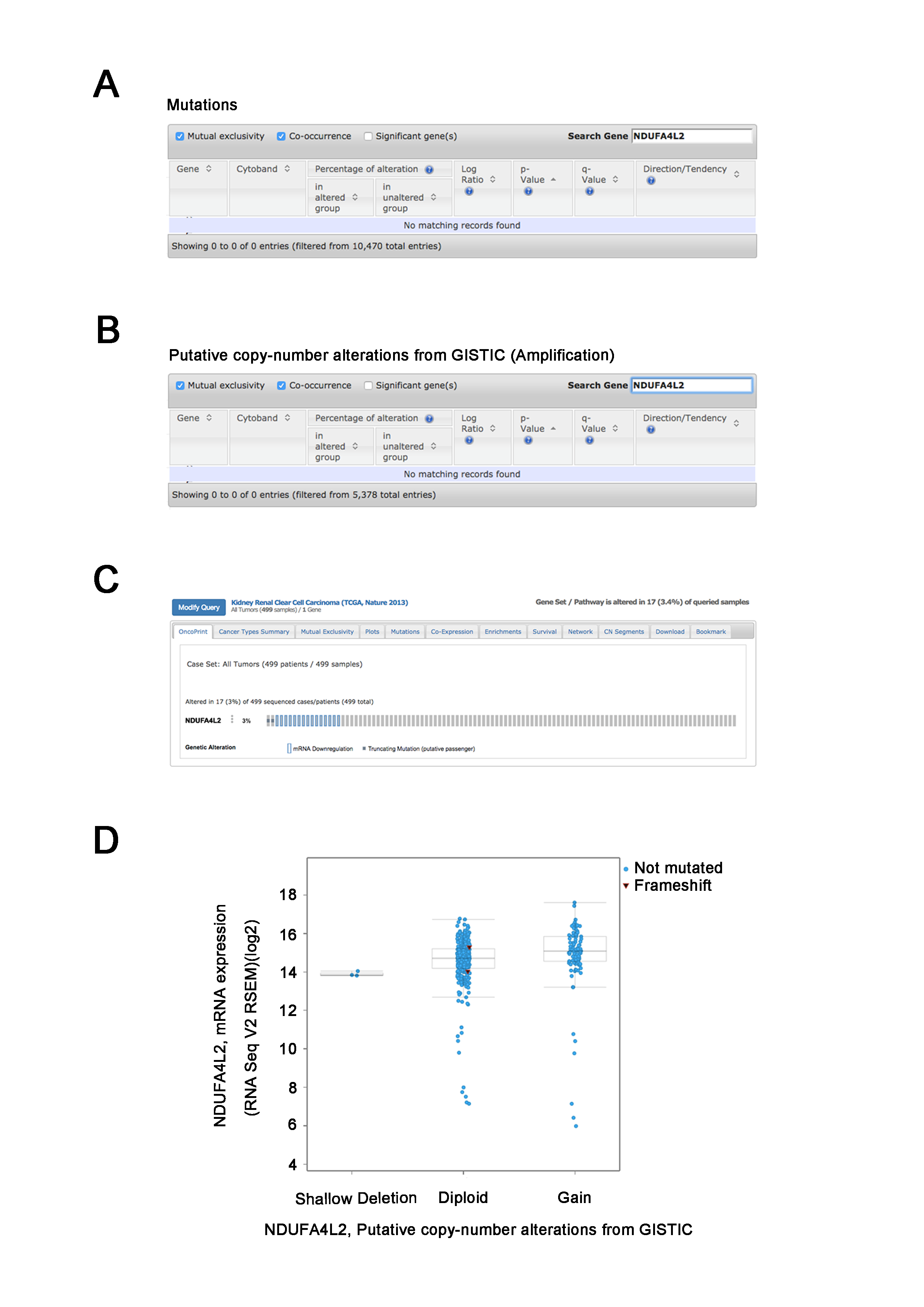

Supplement: Figure S1 — (A–B) Analysis result from cBioPortal TCGA_KIRC dataset showed that there were no mutations or copy number amplification for NDUFA4L2 gene in ccRCC. (C) The OncoPrint from cBioPortal showed that NDUFA4L2 genetic alterations occurred in 3% (17/499) ccRCC cases. However, these genetic alteration of NDUFA4L2 did not lead to the upregulation of NDUFA4L2 mRNA level. (D) Compared with normal copy number samples (diploid), copy number gain samples did not lead to the significant increase of NDUFA4L2 mRNA level. [file peerj-05-4065-s001.png]
